# Testing Design Bioethics Methods: Comparing a Digital Game with a Vignette Survey for Neuroethics Research with Young People

**DOI:** 10.1080/23294515.2022.2110964

**Published:** 2022-08-22

**Authors:** David M. Lyreskog, Gabriela Pavarini, Edward Jacobs, Vanessa Bennett, Geoffrey Mawdsley, Ilina Singh

**Affiliations:** a NEUROSEC, Department of Psychiatry, University of Oxford, Oxford, UK;; b Wellcome Centre for Ethics and Humanities, Oxford, UK;; c Oxford Uehiro Centre for Practical Ethics, Oxford, UK;; d Ethox Centre, Oxford Population Health, University of Oxford, Oxford, UK

**Keywords:** Empirical bioethics, mental health, methodology, design bioethics, digital technologies, games

## Abstract

**Background:**

Over the last decades, the neurosciences, behavioral sciences, and the social sciences have all seen a rapid development of innovative research methods. The field of bioethics, however, has trailed behind in methodological innovation. Despite the so-called “empirical turn” in bioethics, research methodology for project development, data collection and analysis, and dissemination has remained largely restricted to surveys, interviews, and research papers. We have previously argued for a “Design Bioethics” approach to empirical bioethics methodology, which develops purpose-built methods for investigation of bioethical concerns. In this paper we compare a research tool created using a design bioethics approach to a “methods-as-usual” approach in empirical bioethics.

**Methods:**

Our study compared dimensions of engagement with a digital game we created, called “Tracing Tomorrow,” to a standard vignette survey. The two tools investigated the same subject matter, digital phenotyping for mental health, in a sample of 301 UK adolescents.

**Results:**

Participants who played the game reported a greater sense of presence, emotional engagement, cognitive absorption, and mental health ethics insight, compared to participants who completed the vignette survey. Perceived authenticity and curiosity/motivation to learn more was equivalent for both methods.

**Conclusion:**

The results of this study highlights the importance of purpose-built methodology for empirical bioethics research.

## Introduction

With the so called “empirical turn” in bioethics, research into the moral and ethical aspects of daily life has increasingly been carried out using methods and tools from cognate fields to measure and analyze related phenomena (Borry, Schotsmans, and Dierickx 2005). However, as the fields of psychology, neuroscience, and social sciences have enjoyed substantial methodological advancements and experimentation – including digital games and virtual realities (Lange and Pauli 2019), – empirical bioethics has largely stayed within the safe fold of traditional methods: surveys, interviews, vignettes and case studies. While such methods are sufficient for many research purposes, in other cases the research questions and researchers’ epistemological and theoretical commitments require methods typically outside of this range (Earp et al. 2020; Pavarini et al. 2021).

With this in mind we have recently argued for “design bioethics”: the “design and use of purpose-built, engineered tools for bioethics research, education and engagement” (Pavarini et al. 2021). Design Bioethics invites researchers to be methodologically critical, reflexive and innovative. Purpose-built tools, in particular those deploying digital innovations, can help address practical challenges typically faced by traditional methodologies, including scale and reach. The limited use of large and diverse samples in bioethics is problematic, particularly as empirical research designed to inform health policy and practice risks being based on weak, homogenous, and/or non-representative samples. Purpose-built digital tools can also improve inclusion of traditionally hard-to-reach groups, including children and adolescents, and improve interest in participation and retention in research studies.

We have further argued that the use of innovative tools in bioethics enhances researchers’ ability to meet key theoretical commitments in the field. One such commitment is the notion that moral deliberation and decision-making – domains commonly studied in empirical bioethics research – are highly contextual, social, and relational. What we hold to be the morally just thing to do at a given moment, and why we hold it to be so, is intimately embedded in contexts (Patil et al. 2014; Dawson 2013; Kon 2009; Ives 2008).

While some empirical bioethics methods, such as vignette surveys, have been used for many years to capture context dependent data (Ulrich and Ratcliffe 2007), vignettes can only do so much in imitating real-life reactions and simulating vivid representation of moral issues. New and emerging methodologies, such as digital games and world-building narratives, might better capture these key aspects of moral deliberation and decision-making by observing participants in immersive, engaging environments where they make decisions in real time. Such an approach can enable researchers to collect more authentic and valid data; authentic, in the sense that the data is more likely to represent the true values and preferences held by participants, and valid in the sense that the data is relatively unpolluted by distracting elements or unduly priming, or risks issues relating to replicability. As such, these tools might also enhance participants’ insight into bioethical issues and spark motivation to learn more, potentially promoting civic values such as participation in ethically relevant debates. Moreover, such tools also hold the potential to engage people in ways that provoke moral deliberation, opinion building, and relationship building between participants and research and policy communities.

This paper situates within the wider literature on the methods and roles of empirical bioethics (i.e., conceptual, descriptive, and normative) and what it means for empirical bioethics to be sound (Salloch 2021; Salloch, Schildmann and Vollmann 2012; Salloch, Vollmann and Schildmann 2014; Frith 2012). While there are many more stones which need turning before we may begin to make out the frames of this field, we can only do so by – in parallel with conceptual reengineering – diligently and robustly develop, test, and report on tools and methods which make out the space.

In this study we test the claim that a purpose-built game for bioethics research can be used to engage participants with ethically complex topics and simulating vivid scenarios. We compare participants’ experiences of playing a purpose-built “bioethics game” to that of filling in an equivalent vignette-survey. We focused on participants’ sense of presence in the environment created by the game vs. vignette-survey, and cognitive absorption, defined as “a state of deep involvement” (Agarwal and Karahanna 2000). Cognitive absorption encompasses temporal dissociation, immersion, enjoyment, control over the environment and curiosity. We additionally investigated a bioethics game (vs. vignette survey) would lead to greater insight into the issue explored and motivation for further intellectual engagement. Finally, because digital games often involve embodying a character, we also considered it important to measure the extent to which participants in the game vs. survey condition judged their answers to reflect their *own* attitudes and choices. We investigate these questions using Tracing Tomorrow (www.tracingtomorrow.org), an online digital game designed to engage young people in the UK with the topic of digital phenotyping for mental health challenges.

## Materials & methods

### Youth involvement

This study was supported by the NeurOX Young People’s Advisory Group (YPAG), a group of adolescents aged 14-18 years who support research projects in the field of ethics and mental health. Through several in-person group sessions, the YPAG provided significant input into the development of the Tracing Tomorrow game (Lyreskog et al. 2022) and provided interactive advice on the wording of the vignette-survey and whether scenarios were equivalent. The YPAG also offered input into the scales used and wording of the qualitative questions. One YPAG member (Maya Rogers) supported a pilot session in a school setting. She organized access to the school and co-facilitated the session alongside the researchers.

### Preregistration and ethics approval

The study was pre-registered on the Open Science Framework. The data, materials and pre-registration are available at https://osf.io/tr7xc/. The study received ethics approval by the University of Oxford Medical Sciences Interdivisional Medical Ethics Committee (R64008/RE002).

### Sample and recruitment

Sample size was calculated using G-power statistical program, based on power of 0.80 and probability of type I error (α) set at 0.01. To detect a small to medium effect size (d = 0.4) for two-tailed comparisons, a total sample of 296 would be required.

Recruitment was targeted at UK adolescents aged 16 to 18 years. Participants were recruited online via standard and promoted social media posts, as well as through the research group’s network of colleagues and participant lists. During the consenting process, participants were asked to confirm they had not previously played the game “Tracing Tomorrow” (which was disseminated to the public in March 2020) and that they were within our target age range.

In total, 554 participants began the study. To ensure fidelity of the data, timing checks were employed to automatically remove participants who repeatedly answered question sets implausibly quickly. We have also excluded participants who did not complete the qualitative questions, or who did not complete the second part of the survey (post-questionnaires after engaging with survey or game). The game (but not the vignette-survey) condition required participants to open a new tab on their browser, and return to the original online survey platform tab after completing gameplay. However, many participants failed to comply with this requirement and were automatically excluded from the study. This resulted in higher exclusion of participants in the game condition than in the vignette-survey condition. The final sample consisted of 301 participants (103 in the game and 198 in the survey condition).

### Procedure

After filling in the consent form, participants were automatically randomized by the survey platform to one of two task conditions: a bioethics game called “Tracing Tomorrow,” or an equivalent vignette-based survey. The game was referred to during the study as a “graphical journey,” rather than a “game,” to avoid potentially biasing results. After completing the allotted activity, participants were asked to fill in quantitative measures of presence and cognitive absorption during the task, perceived authentic engagement with the task, and their subjectively judged insight and interest in “mental health ethics.” They also responded to qualitative questions regarding their experience of their allotted activity in comparison to previous surveys they had completed. Upon study completion participants received a £10 Amazon voucher.

### Bioethics game methodology

Tracing Tomorrow is a digital game that explores implications of using digital phenotyping for mental health challenges. The game, “Tracing Tomorrow,” was disseminated online for free, took approximately 15-20 minutes to finish, and followed a narrative lined with ethically charged dilemmas and decisions. In the game (see www.tracingtomorrow.org for latest version), a series of morally-valenced choices and dilemmas were provided to the participant in a narrative structure. For each dilemma participants were asked to make decisions about what they would do (e.g., whether to tell their parents/friends about a mental health risk assessment). Throughout the game participants were also provided with opportunities to click on a button for further information relating to the key themes being investigated.

### Vignette survey

**Figure 1:**
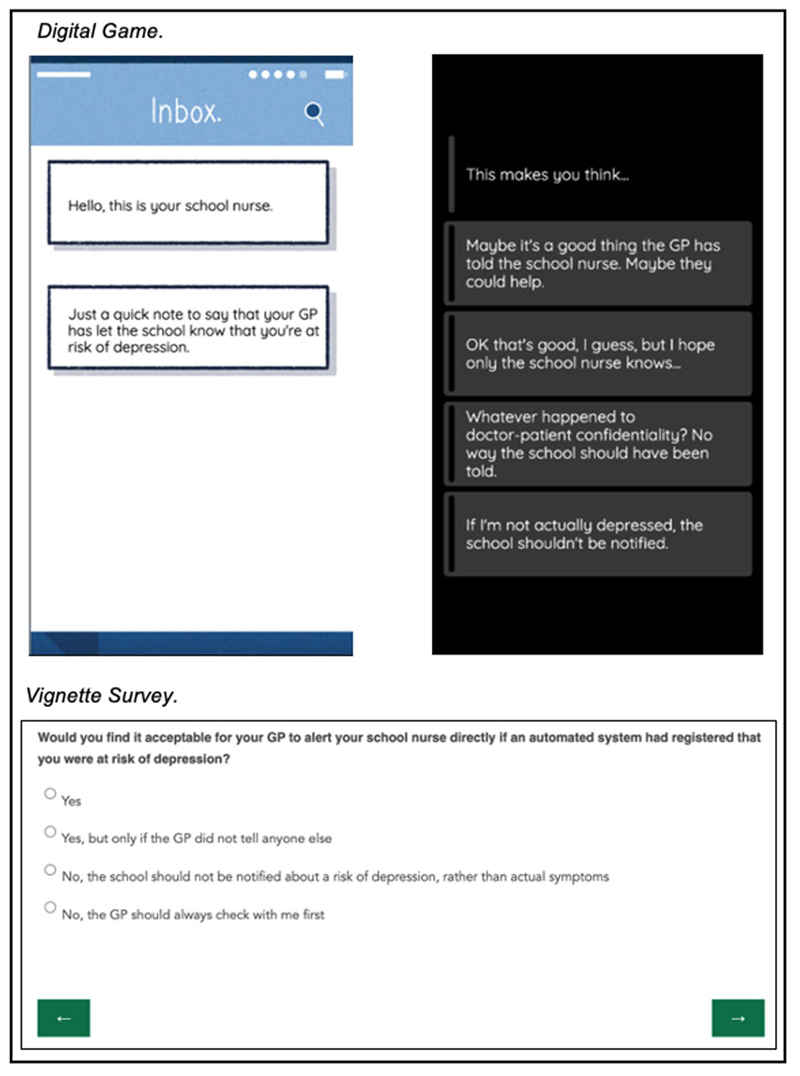
Example question displayed in digital game format and vignette survey format. Created by the authors.

Participants were provided with descriptively rich questions posing dilemmas about mental health tracking technologies and multiple-choice response options in a traditional online survey format. The questions and response options were equivalent to those presented within the game, and they were presented in the same order. However, the questions lacked the detail, interactivity, personalization and narrative continuity (see Figure 1 for a direct comparison).

### Measures

Across all questions, participants were asked to “consider the survey you answered today, in comparison to previous surveys that you have done.” This was to provide a research-relevant anchor and prevent participants from comparing the survey experience with other tasks such as being present in a real-life environment or other types of mediated experience (e.g., virtual reality).

The same 7-point Likert scale was used across all quantitative scales, ranging from “previous surveys a lot more” to “today’s survey a lot more.” The order of the heading was counterbalanced.

### Presence

Participants’ sense of presence was measured by Kim and Biocca’s Presence Scale (Kim and Biocca 1997). The scale comprises eight items measuring two dimensions of presence: *arrival* and *departure*. Arrival refers to the extent to which an individual feels present in the environment (e.g., “At times, the world described by the task was as real or present for me as the ‘real world’”) and departure refers to the extent to which an individual feels they are no longer present in the immediate physical environment (e.g., “During the task, my mind did not enter the world described by the survey, but remained in the room” – reversely coded). The scale had an acceptable level of internal consistency (8 items, α=.655).

### Cognitive absorption

Agarwal and Karahanna’s Cognitive Absorption scale [10] was used to measure this construct’s five dimensions, including: Temporal dissociation (e.g., “Time flew by”) (3 items, α=.767), focused immersion (e.g., “I was absorbed in what I am doing”) (5 items, α=.834), heightened enjoyment (e.g., “I enjoyed it”) (4 items, α=.829), control (e.g., “I felt in control”) (3 items, α=.713) and curiosity (e.g., “It made me curious”) (3 items, α=.765).

### Mental health ethics insight

A five-item scale was created for this study, to measure the extent to which the game/survey provided participants with insights into digital phenotyping for mental health challenges and potential implications. The following items were used, combined into a composite: “It helped me understand the benefits and risks associated with having my data tracked to predict mental health risk,” “It helped me understand how someone’s online activity might give insights into their future risk of mental health challenges,” It made me aware of which people or resources may be able to provide further information about mental health”; “It made it clear what a mental health risk prediction means for someone’s future mental health”; and “It showed me what the impact of receiving a mental health risk prediction may be in my day-to-day life.” The scale had a high level of internal consistency (α=.81).

### Mental health ethics motivation

Using a six-item scale constructed for this study, we measured the extent to which participants felt the game/survey motivated them to learn more, engage in ethically relevant discussions, and contribute to research. The scale encompassed the following items: “It motivated me to learn more about the privacy of online data”; “It made me want to learn more about how new technologies could be used to support someone’s mental health care”; “It inspired me to engage in discussions about the privacy of people’s online data”; “It inspired me to engage in discussions about the role of new technologies on mental health”; “It made me want to contribute to research on the privacy of online data”; “It made me want to contribute to research on the role of new technologies on my mental health.” The scale had a high level of internal consistency (α=.845).

### Perceived authenticity

Four items were constructed to measure the extent to which participants perceived their responses to be an authentic reflection of their values, and how they would behave in real life. Items included: “If faced with these choices in real life, I would choose as I did in this survey”; “My choices in the survey reflected how I would really act”; “My choices in the survey were aligned with who I am as a person”; “The choices I made in the survey represent my own values.” The scale had an excellent level of internal consistency (α=.901).

### Game/survey experience

Participants were asked a number of open questions designed to collectively investigate the way they describe and compare the format of the survey/game with other surveys they had done. These questions were: (1) ‘What did you feel were the key differences between today’s survey and previous surveys you have done, as a means of asking questions?’; (2) ‘How would you describe today’s survey to a friend? Is there anything that sticks out that you will remember? Were there any aspects you particularly liked, or didn’t like?’; and (3) ‘Your last responses suggest that your style of filling in the survey today was a little different compared to other surveys you’ve done. Why do you think that is the case?’. The latter was only displayed to those who indicated the current game or survey was not the same as previously completed surveys, which was assessed through the authenticity scale.’

### Data analysis

Independent samples t-tests were conducted to investigate the effect of condition (2 levels: digital game and digital survey) on the outcome parameters of *presence* (2 subscales), *cognitive absorption* (5 subscales), (perceived) *authenticity, mental health ethics insight*, and *mental health ethics motivation*. As specified in our pre-registration, we set significance levels at 0.01 to account for multiple comparisons.

Open-ended responses were coded using a directed content analysis (Hsieh and Shannon 2005). Because all three questions were meant to elicit similar answers, namely descriptions of salient features of the game/vignette survey compared to traditional surveys, we developed a common coding scheme for the content elicited by all three questions. An initial scheme was developed by GM, with codes informed by relevant theoretical constructs relevant to the study and further iterated and refined to accurately reflect the text data. This initial coding scheme was refined in discussion with DL, GP and EJ based on joint analysis of 25% of the answers. Each of the remaining answers was then coded using the final scheme, and independently coded by a second researcher. Cohen’s Kappa ranged from .65 to .91 across the different codes; reported results are based on the original coder’s results. Each set of answers by each participant was coded dichotomously, based on the presence (1) or absence (0) of different types of content in answers to *either* of the questions within the set. Such dichotomous coding helped control for utterance length, as participants varied in how long their answers were and whether they answered all questions. All coders were blind to conditions.

## Results

### Participants

Participants’ demographic characteristics are displayed in Table 1. Across both groups, most participants were aged 16 or 17 (>80%), self-identified as female (>65%), lived in England (>80%) and attended state comprehensive school (>50%). Both groups were considered ethnically diverse (≤65% White British) relative to the general population (87% White British) (Office of National Statistics 2011). Please note that even though all participants confirmed they were aged 16-18 before starting the study, 10 participants left age blank in the final demographic questionnaire and 2 reported their age as 19. Data analysis without these participants did not alter the magnitude or significance of between-group comparisons; the results we report include the full sample.

### Quantitative analysis

**Figure 2:**
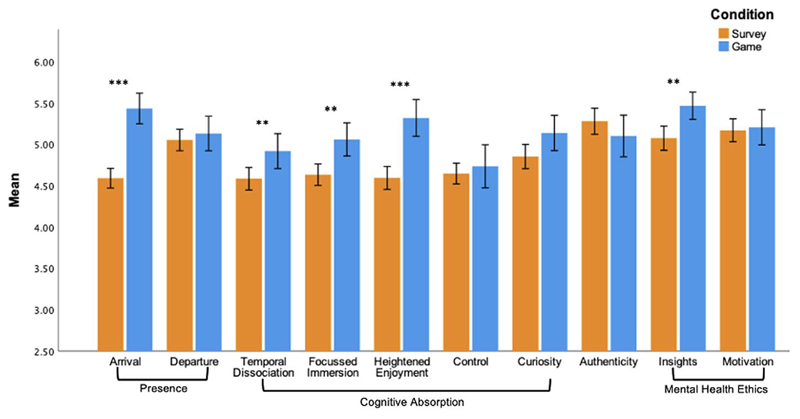
Average scores of presence, cognitive absorption, authenticity and mental health ethics for participants in the game and survey conditions, **p <.01, ***p <.001. Created by the authors.

A summary of mean comparisons between conditions can be found in Figure 2. Participants in the game condition reported a greater degree of presence (“arrival,” where an individual feels more present in their environment), than individuals completing the traditional survey; *t*(299) = −7.701, *p* < .0001, *d* = −.936. No significant difference was observed for presence (departure) (*p* > .05). With regards to cognitive absorption, a significant difference between conditions was found for 3 of the 5 subscales. Compared to those in the survey conditions, participants who played the game reported a greater sense of *temporal dissociation* (*t*(299) = −2.766, *p* = 0.006, *d* = 0.20); a higher sense of *focused immersion* in the environment (*t*(299) = −3.383, *p* = .0008, *d* = 0.20), and greater *heightened enjoyment, t*(299) = −5.448, *p* < .0001, *d* = −.662. Participants’ reported levels of curiosity and sense of control over the environment did not differ between conditions (*p*s > .01).

Compared to the survey, participants in the game condition reported higher levels of *mental health ethics insight*, *t*(299) = −3.267, *p* = .001, *d* = −.397, but equivalent levels of motivation to learn more about the topics covered by the survey (p > .01). Participants perceived their answers as equally authentic across the two conditions (*p* > .01).

### Qualitative results

**Figure 3:**
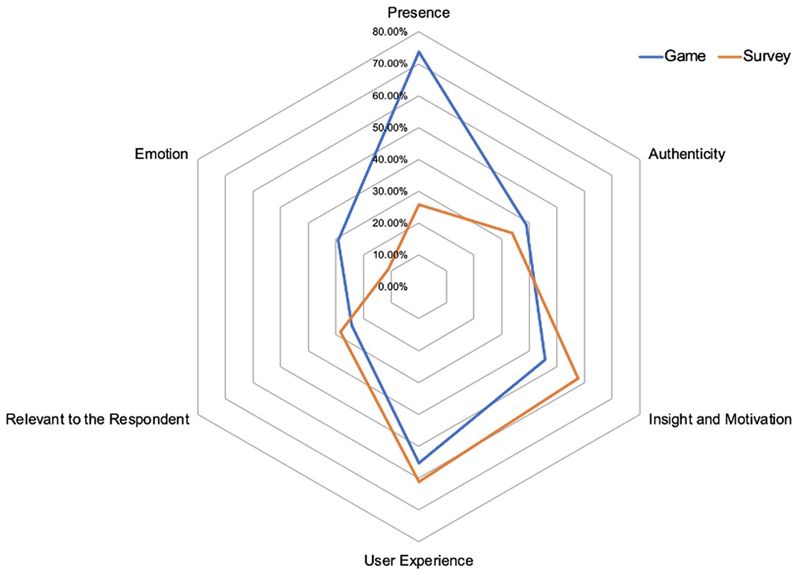
Main codes extracted from open-ended responses reflecting the subjective experience of the digital game vs. vignette survey, and percentage of participants per condition who mentioned each type of content. Created by the authors.

All participants answered the initial two open questions, which asked participants to describe key differences between game/vignette survey and previous surveys, and express how they would describe the game/vignette survey to a friend. A total of 66% of participants in the game condition and 59.6% of those in the vignette survey condition indicated that the game/vignette survey was different from previous surveys they had done, and therefore answered a third open question justifying their choice. Average word count across responses was 17 words. Figure 3 illustrates the main codes identified from participants’ descriptions of their experiences of the game vs. survey in response to the set of open-response questions, and the percentage of participants per condition who mentioned each type of content.

Across both conditions, over half of the participants referred to *User Experience —* that is, practical or technical aspects of the experience such as survey wording, ease of use, comprehensibility and layout. Halima (16), for example, indicated that “today’s survey [was] very easy to understand.” In the game condition specifically this also included references to the graphics and animation. For example, Chloe (16) said “I really liked the animation style and the way you had to scroll to read the next bit. Felt like I was scrolling through Instagram.”

Across both conditions, participants referred to aspects of the method that inspired *Insight and Motivation to Learn*. For instance, participants reflected on the thought-provoking nature of the survey/game and its interesting content (“I would remember the part about data tracking online and what can be done with that data without your knowledge” Lucia, 17, survey). Both methods sparked curiosity to learn more about related topics, for example, “It made me more conscious of how my current data […] and curious as to how safe/secure it would be to try digitally tracking the mental health of people based off […] online activity.” (Irina, 16, survey).

In both conditions a minority alluded to the *Relevance* of the topic to themselves and their lives. For instance, Michael (16) (survey) reported that “the subject of the survey was one that I care deeply about”; similarly for Maria (16) (game) “this survey was more personal and […] made me reflect on myself as a person deeply.”

Participants in both conditions also reflected on the *Authenticity* of their responses. For example Blair (18) (survey) expressed “the situations presented prompted […] more personal answers, rather than what one believes they should” and Lucy reported that “my answers were very genuine to what I would actually choose in real life because I felt I was living it” (Lucy, 16).

In line with quantitative findings, a key difference between conditions in participants’ subjective experience was a heightened level of *Presence* and interactivity in the game condition, compared to the vignette survey. In the game condition participants were three times more likely to allude to aspects of immersion and interactivity. For example, “[The game was] a lot more interactive, which made it really meaningful” (Nia, 18, Game) and “it had a storyline that made me feel immersed” (Isabel, 16). Particularly, the game condition participants reflected on how narrative presence and interactivity facilitated engagement and/or authentic decision-making. For instance, “this survey allowed me to open my mind and put myself in a different world whilst still having more control” (Bashira, 16); “it was very interactive […] my answers were a lot more of an accurate representation of how I would behave*”* (Tishara, 16); “it does feel quite personal (Luke, 16).

In the game condition participants were also more likely to express *Emotional Engagement* or a lasting emotional impact than in the vignette survey condition. For example, “It made me reflect on myself as a person deeply” (Elle, 16) and “It feels more realistic […] I was worried about whether I should accept t + c^1^ at the end” (Alina, 16).

## Discussion

In this study, we compared participants’ experiences of two bioethics methods: a digital narrative game and an equivalent vignette-survey. The study yielded three especially interesting results, consistent across mixed-measures. First, the game produced a significantly higher sense of presence, where participants playing the game were more likely to report that they lost track of time, were less distracted by their surroundings, and felt immersed in the activity. Second, participants reported a heightened level of emotional response to the game, as compared to the vignette survey. Third, participants’ responses on the digital narrative game were perceived as equally authentic as the vignette survey, suggesting that participants were playing as “themselves” rather than embodying a third-person character. Although equally important for a prototype tool like the “Tracing Tomorrow” game, other scores – relating to motivation, user experience, and relevance to participants – were not significantly higher in the game condition than in the vignette survey.

These results indicate that digital games can be built to provide data on values and preferences around bioethical scenarios with emotive and vivid representation. The immersive quality of the game, which secures attention and engagement, represents a cornerstone of our theoretical approach to methods-building. Moral deliberation and decision-making – particularly around complex topics such as “hypothetical digital phenotyping for mental health in schools” – arguably require such faculties to adequately address what is at stake, and how one ought to deal with the ethical issues and dilemmas that emerge (Dewey and Alexander 1998; Walker 1998). Much like immersion and attention, it has been argued that emotional engagement is also key to moral deliberation and decision-making (Mackenzie and Sinnott-Armstrong 2009; Prinz 2009). Despite these advantages in terms of immersion, a key potential concern in using games for research is that the authenticity of responses is at risk, as participants may role play instead of responding from a first-person perspective. With this in mind, our study inquired specifically about the authenticity of responses as perceived by the participants. Notably, this measurement was not designed to determine to what extent participants responded authentically *per se*, but to investigate the prevalence of role playing, and to subsequently help assess its potential indirect impact on authentic choice; while high levels engagement and attention are vital, accompanied by role playing they do not generate authentic representation of choice. However, if role playing levels are low, we come closer to what may be interpreted as authentic engagement. Taken together, our results suggest that narrative digital games can collect data on preferences and values without losing authenticity or accuracy in other key domains.

In addition to supporting moral deliberation, digital games for bioethics research can be preferable to traditional methods for the ability to elicit greater enjoyment, potentially leading to greater scalability. The game we developed and used for our study, *Tracing Tomorrow*, reached 7,337 people in our target population, namely 16-18 year-olds residing in the UK. It was a free game, mainly marketed through social media platforms, with no requirements to play other than disclosure of limited demographic information, and consent to researcher use of anonymised data. The game included a number of “fact buttons” to support informed choice throughout the game, stating facts about mental health, support, and digital phenotyping. Access to adequate information is key to ethical decision-making, and indeed a widely held prerequisite for competent choice The fact buttons in our game were optional (i.e., players did not need to click them to progress); yet almost all players clicked all fact buttons. This suggests that young people have an interest in learning about bioethics and that digital games or other purpose-built bioethics tools can support large-scale engagement and bioethics education. In our study, there was some evidence from the quantitative measure that the game increased participants’ insight into mental health ethics questions.

There is, however, a balance to be struck between engaging participants effectively, while at the same time ensuring valid data is being collected which can be analyzed straightforwardly. A narrative should be engaging and immersive, but must not be leading participants into responding in certain ways for the sake of that narrative, or be phrased in ways which make the responses too difficult to unpack and analyze. This tradeoff between academic accuracy in questions and responses on the one hand, and engagement and dissemination on the other must be carefully assessed. Similarly, a digital game typically immerses participants in highly specific scenarios and narratives, whereas vignettes or surveys are often more open-ended. Whether choices within specific game contexts can be generalized to other similar bioethics scenarios is something that must be addressed in future research.

There are also other potential challenges and pitfalls to be mindful of. Perhaps most prominently, game-based methodologies always involve the risk of roleplaying, leading to non-authentic responses due to participants’ responding through a persona or an avatar. Our game explicitly asked participants to “play as themselves,” and refrained from using characters or avatars on screen to represent the participant, to create a first-person perspective. Future research should investigate whether these cues are necessary or sufficient for authentic moral decision-making. Similarly, there are fine lines to walk in creating immersive and engaging game environments which are representative for diverse populations, and at the same time non-coercive, risking nudging or problematic preconditioning. Finally, extra care needs to be taken in protecting participants’ data if it is collected in a non-controlled digital environment.^2^

## Conclusion

While neuroscience, psychology, and biomedical sciences are constantly developing and improving the research methodologies of their fields, empirical bioethics risks missing out on large-scale and potentially richer data by not utilizing new and emerging tools for data collection. While we have seen that games can improve and engage participants in other domains, it is in no way self-evident that games collecting valid data in an academically rigorous manner can elicit equivalent responses on complex bioethical issues. In this paper we have presented the results of a study of how a digital game, designed to capture young people’s values and preferences in the context of digital phenotyping in schools, compared to a vignette survey with the same purpose. We found that the vignette survey did not do significantly better in any domain, while the game did better in the domains of presence, cognitive absorption and emotional engagement – all important components in moral deliberation. With support in these results, we argue that empirical bioethics research can and should invest in designing theoretically-supported methods for data collection, embracing novel approaches that could support participants’ cognitive and emotional and moral engagement.^2^

## Figures and Tables

**Table 1:** Demographic characteristics. Table created by the authors.

	Game N = 103	Survey N = 198
Age		
16 years	35 (34%)	73 (36.9%)
17 years	50 (48.5%)	91 (46%)
18 years	14 (13.6%)	26 (13.1%)
19 years	1 (1%)	1 (0.5%)
Missing	3 (2.9%)	7 (3.5%)
Gender		
Male	23 (22.3%)	42 (21.2%)
Transgender male	1 (1%)	1 (0.5%)
Female	71 (68.9)	137 (69.2%)
Transgender female	1 (1%)	1 (0.5%)
Non-binary/Other	2 (1.9%)	7 (3.5%)
Prefer not to say	1 (1%)	1 (0.5%)
Uncertain	1 (1%)	4 (2%)
Missing	3 (2.9%)	5 (2.5%)
Ethnicity		
White British	67 (65%)	132 (55.9%)
White Irish/White Other	3 (2.9%)	10 (4.2%)
Black/Black British	4 (3.9%)	7 (3%)
Mixed	7 (6.8%)	14 (5.9%)
Asian/Asian British	16 (15.5%)	22 (9.3%)
Chinese	0 (0%)	2 (0.8%)
Other Ethnic Group	2 (1.9%)	3 (1.3%)
Missing	4 (3.9%)	8 (3.4%)
School		
State comprehensive school	55 (53.4%)	109 (55.1%)
State selective school	21 (20.4%)	48 (24.2%)
Private school	7 (6.8%)	15 (7.6%)
Technical or Technology College	8 (7.8%)	8 (4%)
Other	7 (6.8%)	11 (5.6%)
Missing	5 (4.9%)	7 (3.5%)
Location		
England	89 (86.4%)	177 (89.4%)
Scotland	5 (4.9%)	8 (4%)
Wales	6 (5.8%)	5 (2.5%)
Northern Ireland	1 (1%)	2 (1%)
Missing	2 (1.9%)	6 (3%)

## Data Availability

The data from the quantitative consultation that supported the design of the game is available through the Open Science Framework, at https://osf.io/tr7xc/.

## Footnotes

2 On a more general level, for any research tool developed using a Design Bioethics approach, it is important to be aware of issues in access, capabilities, justice, and other disadvantages which may hinder participation in target populations. Studies are underway to analyse and tackle these issues in Design Bioethics, and in digital mental health in general (See e.g. the EMDYIA project (https://neurogene.org/groups/emdiya/) and the Engajadamente project (https://www.engajada-mente.org/), as future Design Bioethics project must strive to tackle these challenges.

## References

[R1] Agarwal R, Karahanna E (2000). Time flies when you’re having fun: Cognitive absorption and beliefs about information technology usage. MIS Quarterly.

[R2] Borry P, Schotsmans P, Dierickx K (2005). The birth of the empirical turn in bioethics. Bioethics.

[R3] Dawson A (2013). IAB presidential address: Contextual, social, critical: How we ought to think about the future of bioethics. Bioethics.

[R4] Dewey J, Alexander T (1998). The essential Dewey: Ethics, logic, psychology.

[R5] Earp BD, Demaree-Cotton J, Dunn M, Dranseika V, Everett JAC, Feltz A, Geller G, Hannikainen IR, Jansen LA, Knobe J (2020). Experimental philosophical bioethics. AJOB Empirical Bioethics.

[R6] Frith L (2012). Symbiotic empirical ethics: A practical methodology. Bioethics.

[R7] Hsieh HF, Shannon SE (2005). Three approaches to qualitative content analysis. Qualitative Health Research.

[R8] Ives J (2008). ‘Encounters with experience’: Empirical bioethics and the future. Health Care Analysis.

[R9] Kim T, Biocca F (1997). Telepresence via television: Two dimensions of telepresence may have different connections to memory and persuasion. Journal of Computer-Mediated Communication.

[R10] Kon AA (2009). The role of empirical research in bioethics. The American Journal of Bioethics :.

[R11] Lange B, Pauli P (2019). Social anxiety changes the way we move—A social approach-avoidance task in a virtual reality CAVE system. PloS ONE.

[R12] Lyreskog DM, Pavarini G, Lorimer J, Jacobs E, Bennett V, Singh I (2022). How to build a game for empirical bioethics research: The case of ‘Tracing Tomorrow’. Health Expectations.

[R13] Mackenzie C, Sinnott-Armstrong W (2009). Moral psychology, The neuroscience of morality: Emotion, brain disorders, and development.

[R14] Office of National Statistics (2011). Census data.

[R15] Patil I, Cogoni C, Zangrando N, Chittaro L, Silani G (2014). Affective basis of judgment-behavior discrepancy in virtual experiences of moral dilemmas. Social Neuroscience.

[R16] Pavarini G, McMillan R, Robinson A, Singh I (2021). Design bioethics: A theoretical framework and argument for innovation in bioethics research. The American Journal of Bioethics.

[R17] Prinz J (2009). The emotional construction of morals.

[R18] Salloch S (2021). Powers and perils. The American Journal of Bioethics.

[R19] Salloch S, Vollmann J, Schildmann J (2014). Ethics by opinion poll? - The functions of attitudes research for normative deliberations in medical ethics. Journal of Medical Ethics.

[R20] Salloch S, Schildmann J, Vollmann J (2012). The challenge of empirical research in medical ethics: How conceptual accounts on normative-empirical collaboration may improve research practice. BMC Medical Ethics.

[R21] Ulrich CM, Ratcliffe SJ (2007). Empirical methods for bioethics: A primer.

[R22] Walker MU (1998). Moral understandings: A feminist study in ethics.

